# Ovarian development, spawning season, size at maturity and fecundity of *Acentrogobius viridipunctatus* (Valenciennes, 1837) in the Vietnamese Mekong Delta

**DOI:** 10.7717/peerj.14077

**Published:** 2022-09-22

**Authors:** Quang Minh Dinh, Ton Huu Duc Nguyen, Ngon Trong Truong, Diep Xuan Doan, Tien Thi Kieu Nguyen

**Affiliations:** 1 Department of Biology, School of Education, Can Tho University, Can Tho, Vietnam; 2 Department of Molecular Biotechnology, Biotechnology Research and Development Institute, Can Tho University, Can Tho, Vietnam; 3 Medicinal Chemistry, Hi-tech Agriculture & Bioactive Compounds Research Group, School of Engineering and Technology, Van Lang University, Ho Chi Minh City, Vietnam; 4 Department of Biology, An Khanh High School, Can Tho, Vietnam

**Keywords:** Multi-spawner, Ovary, Size at first maturity, Spotted green goby

## Abstract

This present study provides an overview of the reproductive traits, including ovarian development, spawning season, size at maturity (*L_m_*), and fecundity of *Acentrogobius viridipunctatus* – a high economic value fish in the Vietnamese Mekong Delta (VMD). A total of 432 females were collected using trawl nets from January to December 2020 at four sites, including Long Huu-Tra Vinh (LHTV), Trung Binh-Soc Trang (TBST), Dien Hai-Bac Lieu (DHBL), and Tan Thuan-Ca Mau (TTCM). The ovarian diameter was seen to increase from 2.09 (stage I) to 6.57 mm (stage V). Histological analysis showed that the goby was a multi-spawner due to the different oocyte stages found in stages IV and V of ovaries. It can release eggs all year-round due to the monthly appearance of ovarian stages IV and V during the study period, with the main peak in the 2–4 months of the wet season noted with the highest value of gonadosomatic index at that time. The *L_m_* increased from 6.6 cm at TBST to 9.4 cm at TTCM. This species displayed relatively high fecundity, ranging from 5,481 to 130,683 eggs/female. The average fecundity of this fish was 27,698 ± 7,983 eggs/female at LHTV, 46,592 ± 7,264 eggs/female at TBST, 23,271 ± 4,985 eggs/female at DHBL, and 31,408 ± 2,515 eggs/female at TTCM. Egg diameter ranged from 0.45 ± 0.01 at DHBL to 0.50 ± 0.01 at TBST. For sustainable exploitation, local governments should ask the fishers to avoid catching fish during the main spawning period, and the fish length at first capture should be >*L_m_*.

## Introduction

The Vietnamese Mekong Delta (VMD) is the third-largest delta globally (Coleman & Huh, 2012). The VMD is the downstream area of the Mekong River system and is adjacent to the sea. With more than 700 km of coastline and an interlaced river system, the VMD is the preferable place for exploiting and cultivating fish ranging from freshwater to saltwater (Le et al., 2006). In addition, the fish communities that live here are diverse, with 77 families and 322 recorded fish species (Tran et al., 2013). Of these, there are ~80 fish species with high economic value (Thai, 2015). However, the overexploitation of economically valuable fish species has led to a rapid decrease in their population. There has also been the failure of appropriate measures to conserve and protect endangered fish species (Thai et al., 2012). As such, it is necessary to study their biological characteristics in able to develop a suitable strategy for improved fish resources.

Reproduction is necessary for a species to survive and develop (Mai et al., 1979). This is a complex process that involves gonadal development and fish behavior. Changes in the histological characteristics of gonads through different stages aid a better understanding of fish’s reproductive cycle and patterns (Pham & Tran, 2004). In most fish, the gonads are composed of two tubular chambers (Mai et al., 1979). In addition, reproductive biology also plays a vital role in conserving fish species. The length at first maturity (*L*_*m*_) helps recommend appropriate fishing length and is an essential indicator in managing fish stocks (Fontoura, Braun & Milani, 2009; Teichert et al., 2014). Furthermore, the gonadosomatic index (GSI) is helpful in determining the fish spawning season (Plaza et al., 2007; Dinh & Le, 2017). These reproductive biology features are closely related to the production and capture of aquatic species (Miller, 1984; Komolafe & Arawomo, 2007).

Goby is one of the fish groups with high economic and nutritional value in the VMD (Nguyen, 2000), living in both freshwater to saltwater environments (Tran et al., 2013). *Acentrogobius viridipunctatus* is a typical fish of this fish group and has prominent features such as a dark curved line under its eyes and many bright blue spots on its head and body (Tran et al., 2013). This fish is relatively small, with a maximum length recorded in estuarine Israel of 16.5 cm (Bauchot, Ridet & Diagne, 1989). With their modest size, their food source is small animals such as other fish, shrimp, and organic humus (Le & Tong, 2011). However, there is scarce data on this species in the VMD, particularly the reproductive biology of females. Also, along with several other fish species, this species is at risk of habitat loss and overfishing (Thai et al., 2012). Research on the reproductive characteristics of the fish, *e.g*., ovarian development, spawning season and pattern, and length at first maturity, plays an essential role in the raising and artificial reproduction of this fish and may provide insights for a sustainable exploitation strategy.

## Materials and Methods

**Figure 1:**
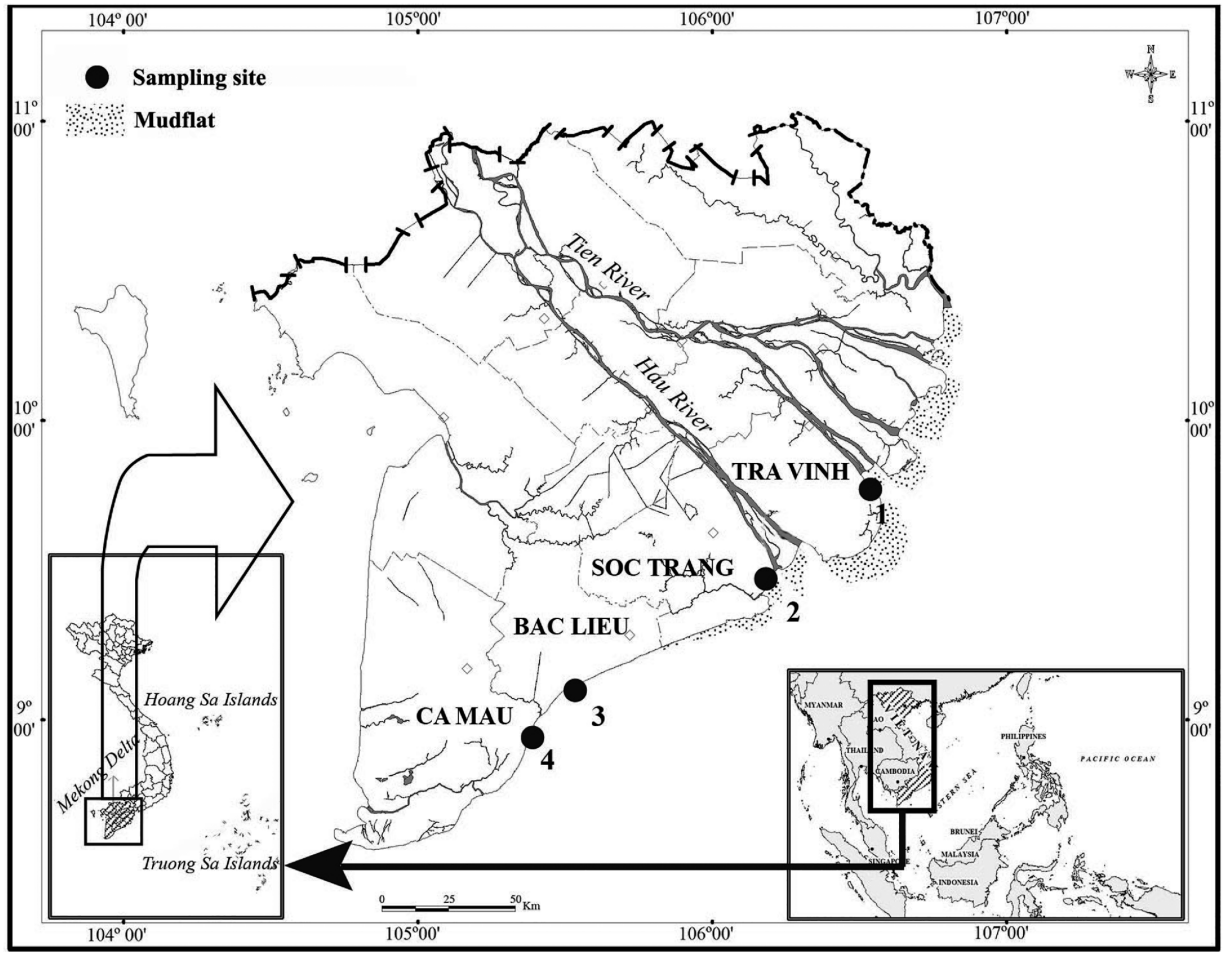
Map of sampling sites. 1: Long Huu-Tra Vinh; 2: Trung Binh-Soc Trang; 3: Dien Hai-Bac Lieu; 4: Tan Thuan-Ca Mau; Dinh (2018).

As *A. viridipunctatus* is distributed mainly in brackish water, the sampling was carried out in estuarine areas where they occur dominantly, such as Long Huu-Tra Vinh (LHTV), Trung Binh-Tran De (TBST), Dien Hai-Bac Lieu (DHBL) and Tan Thuan-Ca Mau (TTCM) (Fig. 1). Here the dry season was from January to May (with no rain), and the wet season was from June to December (with heavy rain) (Le et al., 2006). Fish were collected as previously described in Dinh et al. (2022a). Specifically, fish were collected once a month using trawl nets and were anesthetized with MS222 (25 mg MS22 was diluted with 5 liters of water taken from the sampling site) for ~5 min before fixing in 5% formalin and shifting to the laboratory for analysis. The use of fish was approved by the Scientific Committee of the School of Education, Can Tho University, under the Animal Welfare Assessment number Q2020-01/KSP. According to Dinh et al. (2016), after sampling, the fish samples were sexed using the genital spines between males (pointed triangles) and females (oval shapes). The temperature and salinity of these sites were also recorded using a temperature meter (HI98128) and a Refractometer (SLI-10), respectively.

After measuring total length (TL, in cm) and weight (W, in g), the fish was dissected to remove the ovary for morphological and histological analysis in the laboratory (Animal Laboratory, Department of Biology, School of Education, Can Tho University). The ovary was visually classified into five stages (immature in stage I to maturing in stage II and III, mature in stage IV, ripe in stage V) of development using the criteria description for *Parapocryptes serperaster*, documented by Dinh et al. (2016). The ovary was then weighed to the nearest 0.1 mg using a precision balance and measured in diameter to the nearest 0.01 mm using a Motic Image-Pro Plus v.2.0 integrated with the stereomicroscope. Thereafter, 25 ovaries (five samples for each stage) were selected to examine histologically using the staining process of Ho, Nguyen & Dinh (2021), including fixing, dehydrating, paraffin wax embedding impregnating, 6-μm thick cutting and staining with Hematoxylin and Eosin-Y, for gamete developmental determination.

The length at first maturity (*L*_*m*_) was the length in which 50% of fish reached sexual maturity and calculated using the formula P = 1/(1 + e^−r(*TL − Lm*)^) (King, 2013), where P is the percentage of adult fish (%); and TL is the total length of the fish (cm).

The spawning season was determined based on ovarian frequency composition and the gonadosomatic index (GSI) (Plaza et al., 2007; Dinh & Le, 2017). Whereas, the GSI was calculated as GW * 100/W (GW, 0.1 mg) (Lloret & Rätz, 2000).

A total of 60 ovarian stages IV onwards (15 samples per site) were used to assess batch fecundity. The ovaries were soaked in water to prevent oocyte rupture, and a pen was used to remove the membranes and separate oocytes. The oocytes were observed and counted under a Motic stereomicroscope to determine the exact number and diameter of eggs (Nguyen et al., 2021). According to Bagenal (1967), batch fecundity was from the formula: *F = (n* × *G)/g* (*F*: batch fecundity; *n*: the number of oocytes in sub-sample; *g*: the weight of sub-sample; and *G*: the ovarian weight). The oocyte diameter per ovarian stage, according to Dinh et al. (2022c), was determined by randomly measuring 30 oocytes in each ovary using Motic Image-Pro Plus v.2.0.

One-way ANOVA with Tukey *Post-hoc* test determined the monthly variation of the GSI and spatial change of salinity and temperature at a 5% significance level. According to the method of Morey et al. (2003), the t-test was used to verify if the *L*_*m*_ varied significantly between sites at a *p*-value < 0.05. SPSS v.21 software was used for statistical processing. The total length, weight, and batch fecundity values were transformed into the log10 before being used to qualify the relationship between fecundity with length and weight *via* linear regression.

## Result

### Environmental characteristics at the study sites

According to the study site, measurement results over 12 months showed a significant difference in salinity, with salinity reaching the highest value at DHBL (32.3 ± 1.1‰) followed by TTCM (28.7 ± 1.1‰) and lowest at LHTV (17.8 ± 2.3‰) and at TBST (19.8 ± 2.4‰) (One-way ANOVA, F = 15.73, df = 3, *p* < 0.001) (Table 1). However, the temperature did not differ over the study sites and ranged from 30.1 ± 0.5 °C in TTCM to 31.0 ± 0.4 °C in LHTV (F = 0.99, df = 3, *p* = 0.40) (Table 1).

**Table 1:** Measured temperature and salinity at the four sampling sites.

Month	Long Huu-Tra Vinh		Trung Binh-Soc Trang		Dien Hai-Bac Lieu		Tan Thuan-Ca Mau	
	Temperature (°C)	Salinity (‰)	Temperature (°C)	Salinity (‰)	Temperature (°C)	Salinity (‰)	Temperature (°C)	Salinity (‰)
01/2020	28.3	23.0	29.2	20.0	28.0	25.0	26.5	24.0
02/2020	29.3	20.0	27.9	25.0	27.5	32.0	28.0	30.0
03/2020	28.8	27.0	26.3	29.0	27.1	28.0	28.3	31.0
04/2020	31.0	18.0	30.8	19.0	30.1	32.0	30.1	28.7
05/2020	31.0	28.0	30.1	31.0	31.7	34.0	32.0	32.0
06/2020	31.8	14.0	32.3	19.0	30.3	37.0	30.6	33.0
07/2020	32.0	10.0	33.3	13.0	31.2	33.0	30.7	27.0
08/2020	32.1	12.0	31.9	10.0	31.0	32.0	30.9	25.0
09/2020	32.2	9.0	33.1	10.0	30.9	31.0	30.7	24.0
10/2020	32.5	29.0	30.7	32.0	32.3	35.0	32.6	33.0
11/2020	31.9	9.0	32.8	10.0	30.6	31.0	30.4	24.0
12/2020	31.5	14.0	32.0	19.0	30.0	37.0	30.3	33.0
Mean	31.0	17.8	30.9	19.8	30.1	32.3	30.1	28.7
SE	0.4	2.3	0.7	2.4	0.5	1.0	0.5	1.1

### Ovary development and spawning pattern

A total of 960 individuals (528 males and 432 females) were sampled at these sites from January to December 2020. Of them, 432 females were used in this study. Hereafter the number of males was used in another study (Table 2). Analytical results showed that the ovary of *A. viridipunctatus* was long, tubular, and composed of two chambers located close to the spine in the abdominal cavity. Connective membranes fixed the outside of the fish ovary. However, the ovary showed different morphological and histological features through each stage. Specifically,

**Table 2:** Total length (TL) and weight (W) of fish at sampling sites for 12 months.

Month	Long Huu – Tra Vinh			Trung Binh – Soc Trang			Dien Hai – Bac Lieu			TTCM		
	Total	W_min_–W_max_	TL_min_–TL_max_	Total	W_min_–W_max_	TL_min_–TL_max_	Total	W_min_–W_max_	TL_min_–TL_max_	Total	W_min_–W_max_	TL_min_–TL_max_
01/20	5	4.59–13.63	7.3–10.1	6	5.24–14.48	6.6–10.7	3	4.36–19.45	6.5–10.5	14	6.19–13.68	7.2–10.7
02/20	14	4.28–7.00	6.8–8.2	5	5.56–13.63	7.1–10.1	9	6.00–23.91	7.6–11.5	11	9.49–23.41	9.6–12.6
03/20	7	4.26–13.70	7.0–9.5	4	5.56–13.09	7.1–9.9	16	5.38–19.80	6.8–11.5	3	5.13–22.88	7.6–12.4
04/20	18	6.53–23.13	8.2–12.2	8	9.23–15.99	8.6–11.6	11	4.24–10.04	6.3–8.9	12	6.33–28.13	8.0–13.5
05/20	4	11.92–17.17	9.6–10.8	13	2.47–34.25	5.9–13.1	14	5.02–13.29	6.8–9.9	10	8.36–23.29	8.5–12.5
06/20	12	7.28–16.09	8.5–11.2	2	10.12–25.36	9.7–13.6	7	10.86–18.7	10.1–11.4	10	6.79–20.85	7.8–11.9
07/20	11	9.40–17.62	8.5–11.8	6	9.65–14.82	9.7–10.6	5	5.09–18.81	7.2–11.2	13	5.88–16.16	7.2–11.0
08/20	13	4.30–19.23	7.0–11.5	5	6.33–24.88	7.6–13.6	3	6.17–16.63	7.4–12.6	12	6.88–12.02	7.5–10.5
09/20	6	4.75–11.70	6.5–9.0	16	4.50–21.75	6.5–11.4	3	6.93–16.03	8.6–11.9	12	5.72–25.20	7.2–11.6
10/20	7	8.56–13.46	8.2–10.0	8	5.40–8.29	8.6–10.4	15	7.20–22.10	8.7–12.6	10	4.99–11.81	7.4–10.1
11/20	7	7.92–14.83	8.4–10.5	11	5.01–8.24	7.4–9.6	3	6.54–15.40	8.3–11.6	13	6.73–15.84	8.2–12.0
12/20	5	4.19–7.18	7.0–8.8	9	4.9–16.12	7.2–11.2	3	6.54–15.12	8.3–11.2	18	5.90–25.78	7.6–12.8
Total	109	4.19–23.13	6.5–12.2	93	2.47–34.25	5.9–13.6	92	4.24–23.91	6.3–12.6	138	4.99–28.13	7.2–13.5

**Figure 2:**
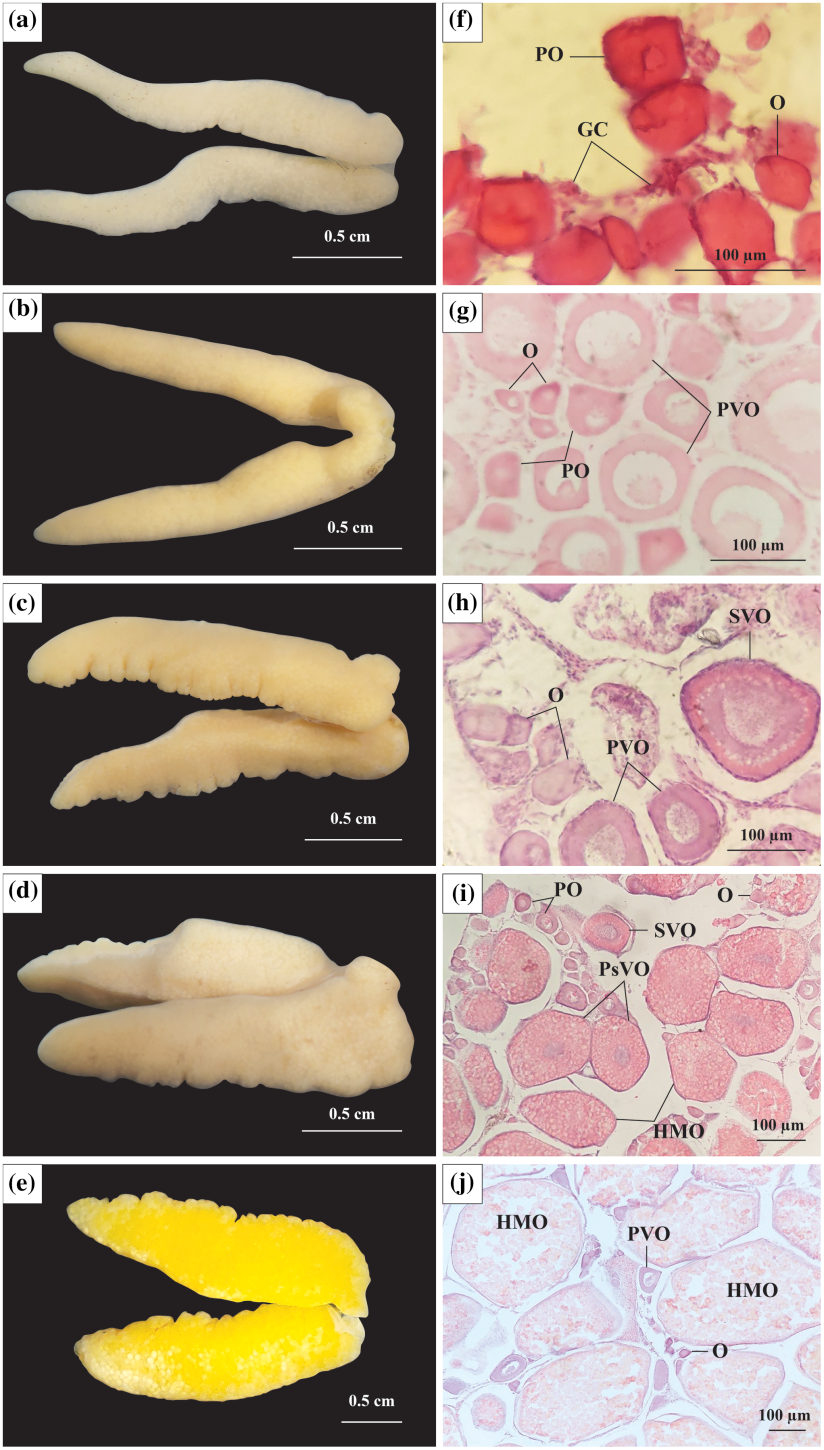
The morphology and histological in the ovary of *Acentrogobius viridipunctatus*. (A–E) Stage I–V of the ovary; (F–J): histology of the ovary in stages I–V; GC, germ cells; O, oogonia; PO, primary oocyte; PVO, primary vitellogenic oocytes; SVO, secondary vitellogenic oocytes; PsVO, post vitellogenic oocytes; HMO, hydrated oocytes; sampled from Tan Thuan-Ca Mau.

Stage I: At this stage, the ovary was observed to be milky white, thin in size, and had an average diameter in 21 ovaries of 2.09 mm (Fig. 2A). The ovarian histological structure consisted of germ cells (GC) and oogonia (O). In addition to the GC and O, several primary oocytes (PO) were scattered throughout the ovary. The GC was small, had large nuclei, and was located in clusters, whereas O was more significant in size, acquired a dark purple colour with Hematoxylin, and divided. The PO grew from O and had a central nucleus. At this stage, yolk sacs did not appear (Fig. 2F).

Stage II: At this stage, the ovary increased to 2.47 mm (45 ovaries) in diameter and was light yellow in colour (Fig. 2B). The PO appeared predominantly, and the number of O gradually decreased, leaving only a few clusters distributed interspersed with GC. Some PO was generated before dividing into primary vitellogenic oocytes (PVO). The nuclear rings were found in PO and PVO (pale with Hemtaoxyline cells) (Fig. 2G).

Stage III: The ovary diameter at this stage was 3.19 mm (83 ovaries), and the surface was rough and wavy (Fig. 2C). In the ovary, most of the PO had developed to PVO. The PVO further developed into secondary vitellogenic oocytes (SVO) with numerous yolk sacs containing yolk granules. At this stage, the oocyte developed strongly in cytoplasmic size. The ratio between nuclear volume and cell volume during this period was seen to be significantly reduced, yet the O still appeared during this period in small numbers. At the end of this phase, half of the cytoplasm located near the periphery was filled with yolk sacs (Fig. 2H).

Stage IV: Ovary occupied 1/2 volume of the abdominal cavity and reached 4.05 mm (250 ovaries) in diameter (Fig. 2D). The ovary had the addition of post vitellogenic oocytes (PsVO) and hydrated oocytes (HMO). The size of the oocyte was almost at its maximum, containing high nutrients. During this stage, the PsVO and HMO occupied most of the area in the ovary. The HMO was spherical pale pink, occupying most of the volume. The oocyte’s nucleus began to contract, the nuclear membrane gradually disappeared, and most nuclei moved to the nucleus’ centre (Fig. 2I).

Stage V: The size of the ovary reached its largest size with a diameter of 6.57 mm (33 ovaries) and bright yellow in colour (Fig. 2E). Each egg can be observed from the outside when pressed firmly against the fish belly. The histological structure at this stage was mainly HMO. In addition, in the cross-sectional smear of the ovary, GC regions were also observed. This was the basis for the continued development of the ovary (Fig. 2J).

### Spawning season

**Figure 3:**
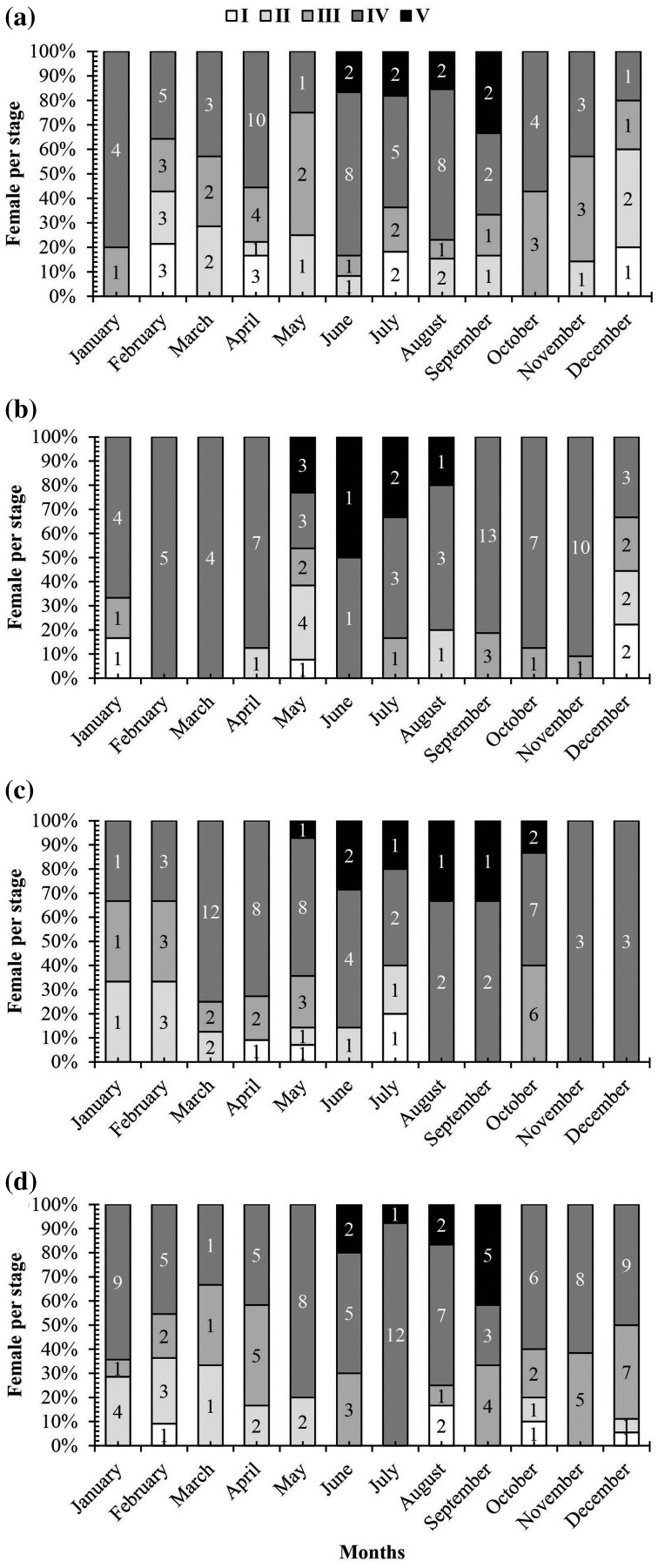
The ovarian frequency composition of *Acentrogobius viridipunctatus*. (A) Long Huu-Tra Vinh, (B) Trung Binh-Soc Trang, (C) Dien Hai-Bac Lieu, (D) Tan Thuan-Ca Mau; number in each column: number of individuals.

**Figure 4:**
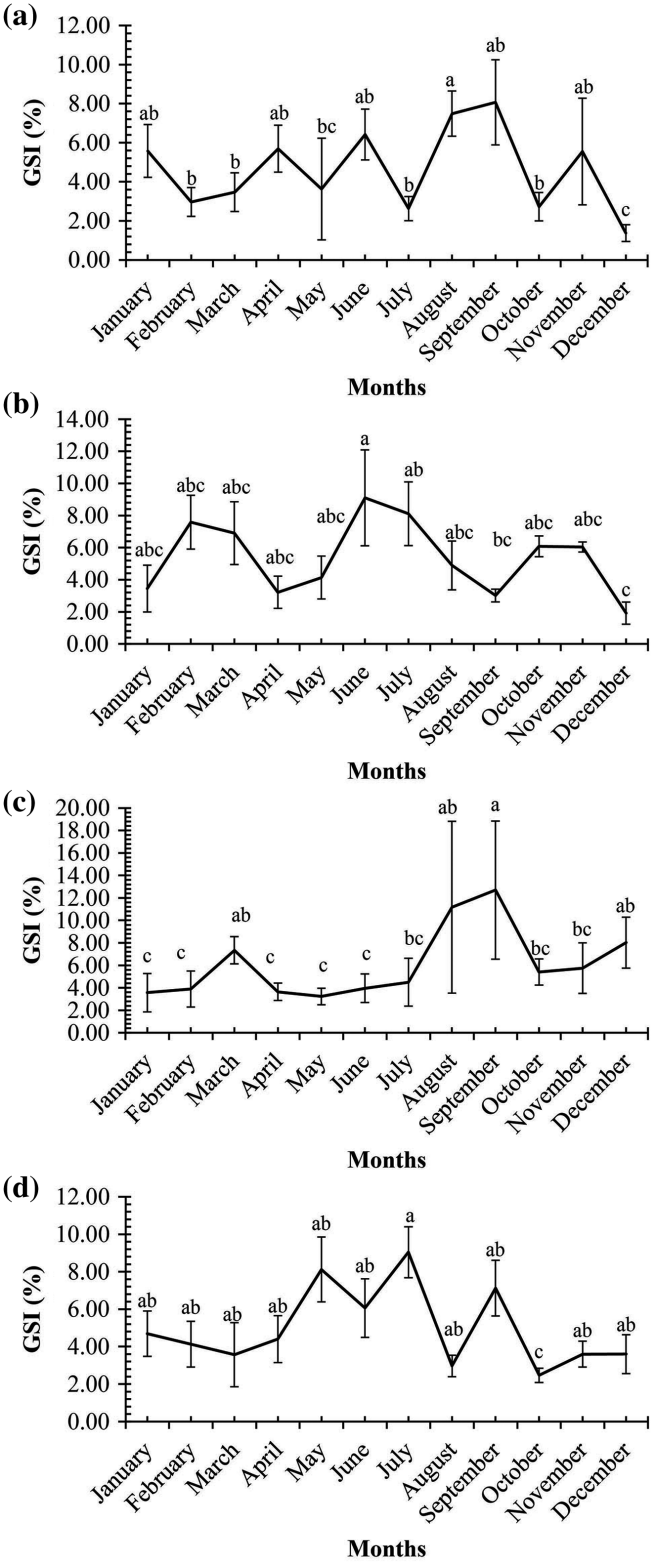
Gonadosomatic index of *Acentrogobius viridipunctatus*. (A) Long Huu-Tra Vinh, (B) Trung Binh-Soc Trang, (C) Dien Hai-Bac Lieu, (D) Tan Thuan-Ca Mau; different letters denote the variation in the monthly GSI values at each site.

A graph of the maturation stages of *A. viridipunctatus* showed the number of individuals obtained per month and the stage of ovary development in this species (Fig. 3). Each color in each column represents the ratio of a different stage, and the number in each box represented the number of individuals at that stage. Analysis of the frequency of occurrence of the ovary at the four study sites for the 12 months revealed that this fish belongs to the group of fish that reproduce many times during the spawning season. Ovarian stages IV to V were found at all months (Fig. 3). To know the exact breeding season of fish, it was necessary to rely on the change in the value of GSI between months. At LHTV, the GSI of this fish had reached its highest value from August to September (One-way ANOVA, df = 11, F = 2.22, *p* = 0.02, Fig. 4A). Meanwhile, in TBST, the highest value of GSI only appeared in the months of June and July (F = 3.28, df = 11, *p* = 0.001, Fig. 4B). Similarly, at DHBL, GSI displayed high values in August and September (F = 1.98, df = 11, *p* = 0.04, Fig. 4C). Finally, at TTCM, the period from May to July was the time with the highest GSI value (F = 2.99, df = 11, *p* = 0.001, Fig. 4D). Although the change in GSI was seen across study sites, this species generally has a highly concentrated spawning season in the wet season.

### Length at first maturity and fecundity

**Figure 5:**
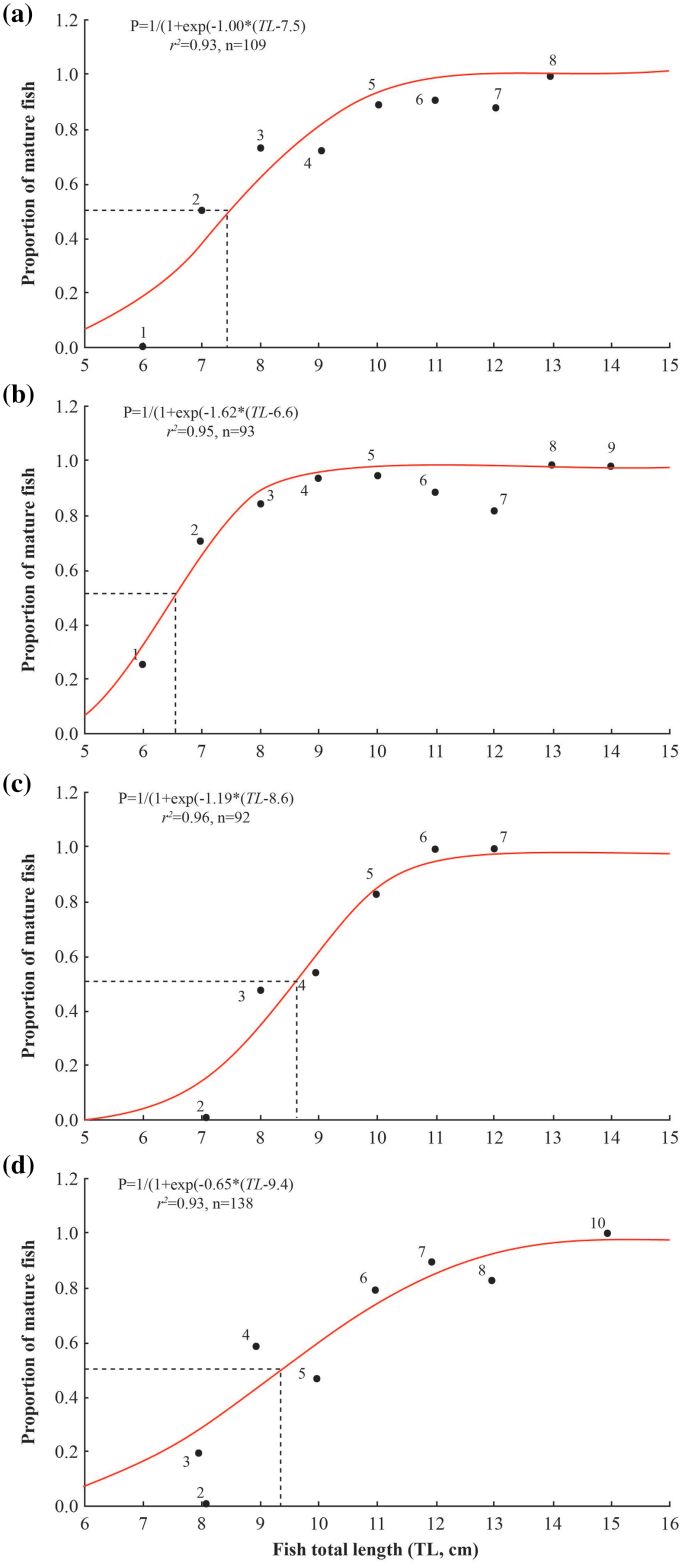
Size at first maturity of *Acentrogobius viridipunctatus*. (A) Long Huu-Tra Vinh, (B) Trung Binh-Soc Trang, (C) Dien Hai-Bac Lieu, (D) Tan Thuan-Ca Mau.

The length at first maturity of *A. viridipunctatus* varied between the four study sites, reaching the highest value at TTCM (9.4 ± 0.4 SE), followed by DHBL (8.6 ± 0.2 SE) and LHTV (7.5 ± 0.3 SE), and the lowest value at TBST (6.6 ± 0.2 SE) (Fig. 5). Specifically, *L*_*m*_ at TBST was significantly lower than that at LVTH (t = 4.50, df = 16, *p* < 0.001), DHBL (t = 10, df = 2, *p* = 0.01), and TTCM (t = 14, df = 45, *p* < 0.001). The *L*_*m*_ at LHTV was significantly lower than that at DHBL (t = 3.67, *p* = 0.002) and TTCM (t = 6.33, df = 29, *p* < 0.001). Similarly, the *L*_*m*_ of DHBL was significantly lower than that of TTCM (t = 4.00, df = 46, *p* < 0.001).

**Figure 6:**
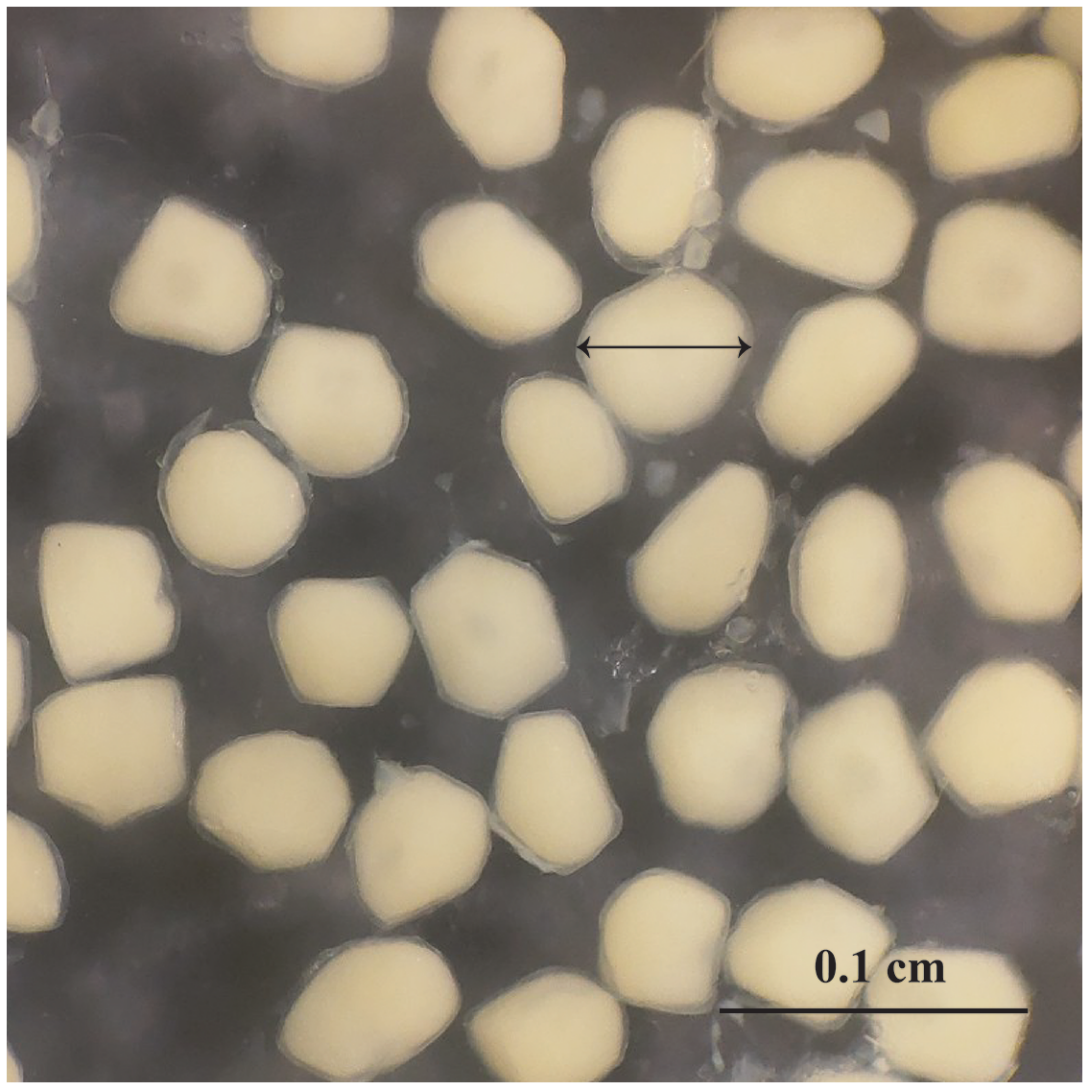
The outer appearance of the egg in *Acentrogobius viridipunctatus*. Sampled from Tan Thuan-Ca Mau.

**Figure 7:**
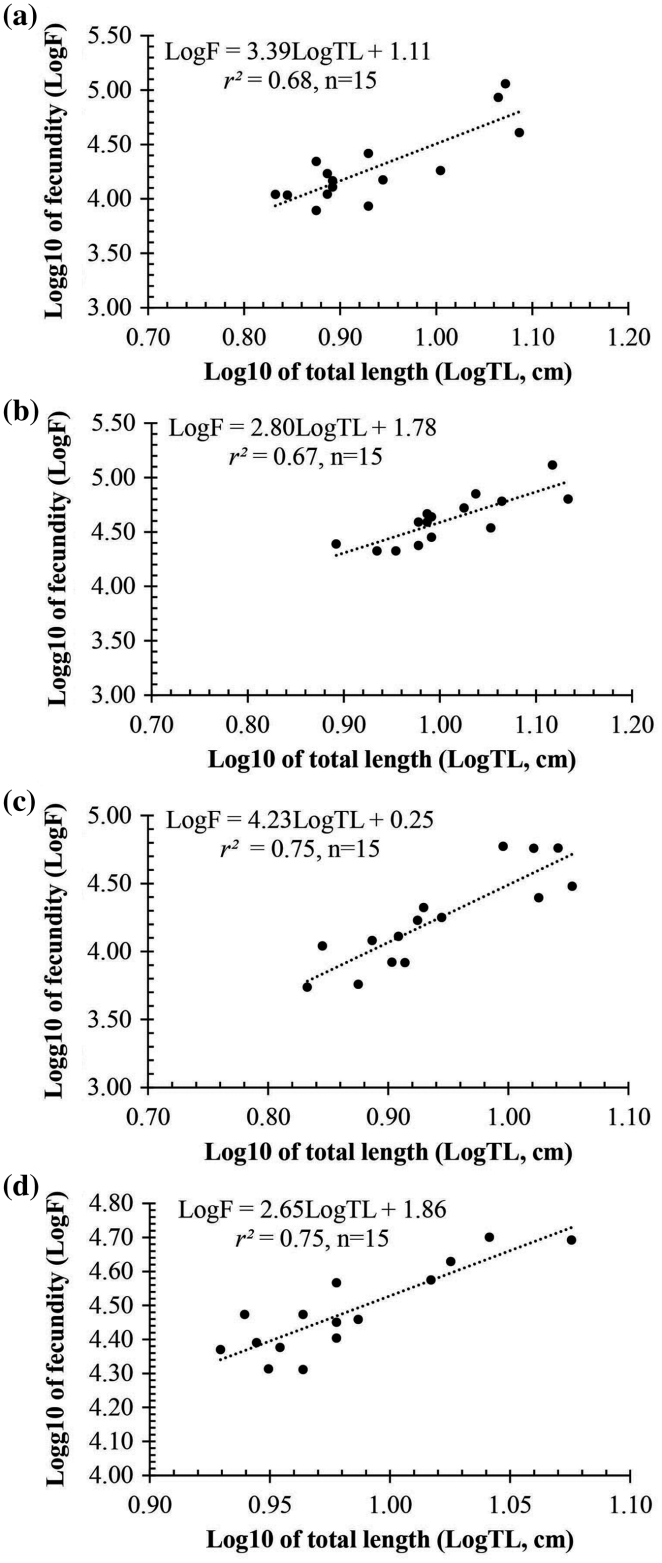
The relationship between fecundity with the total length of *Acentrogobius viridipunctatus* at study sites. (A) Long Huu-Tra Vinh, (B) Trung Binh-Soc Trang, (C) Dien Hai-Bac Lieu, (D) Tan Thuan-Ca Mau.

**Figure 8:**
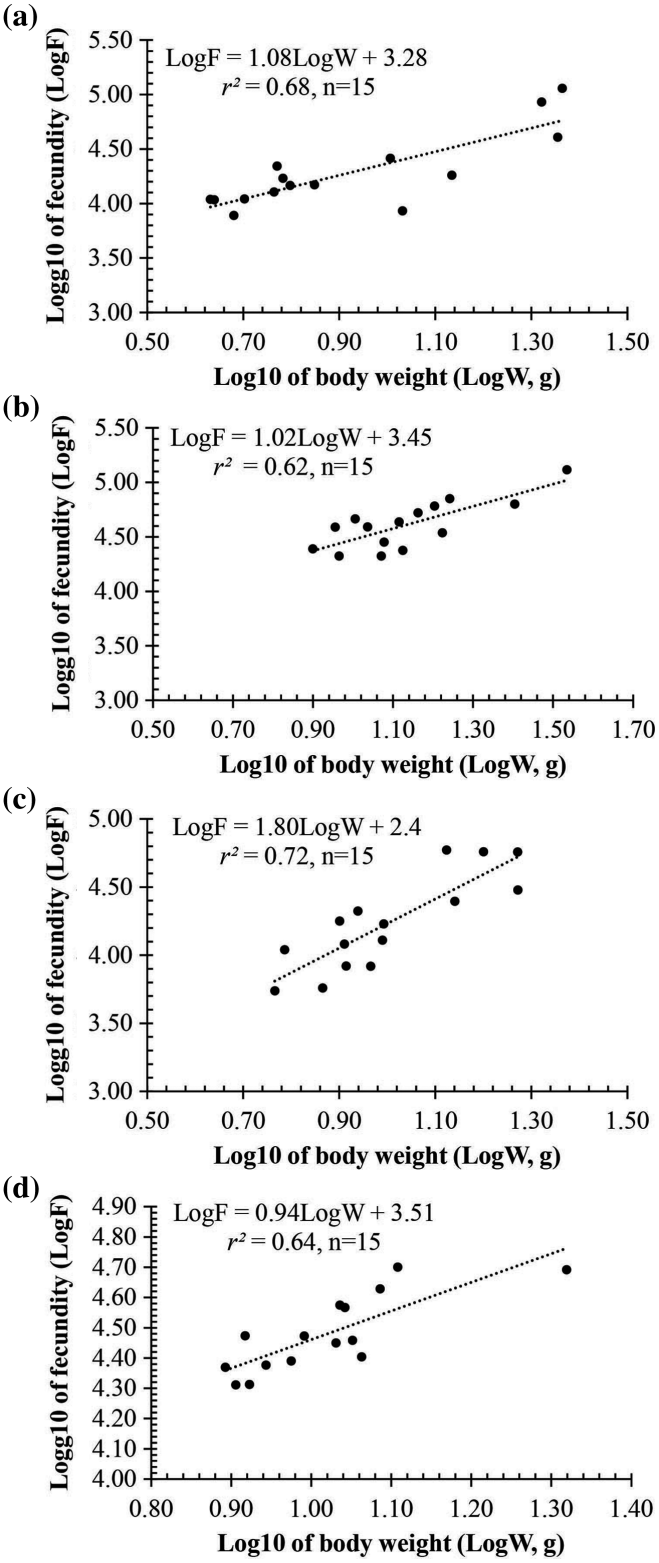
The relationship between fecundity with the body weight of *Acentrogobius viridipunctatus* at study sites. (A) Long Huu-Tra Vinh, (B) Trung Binh-Soc Trang, (C) Dien Hai-Bac Lieu, (D) Tan Thuan-Ca Mau.

Observation results under the stereomicroscope showed that the HMO (*e.g*., egg) of *A. viridipunctatus* was spherical (Fig. 6). The mean egg diameter varied between the four study sites. The fecundity *of A. viridipunctatus* was quite high (5,481–130,683 eggs/female) and varied from site to site. Similarly, this fish also exhibited the highest value of *F* at TBST (46,592 ± 7,264 eggs/female, *n* = 15) and the lowest at DHBL (23,271 ± 4,985 eggs/female, *n* = 15). The F value of the two remaining sites was 27,698 ± 7,983 eggs/female (*n* = 15) at LHTV and 31,408 ± 2,515 eggs/female (*n* = 15) at TTCM. In this fish, fecundity was seen to be closely related to total fish length (*r*^*2*^_LHTV_ = 0.68; *r*^*2*^_TBST_ = 0.67; *r*^*2*^_DHBL_ = 0.75; *r*^*2*^_TTCM_ = 0.75; Fig. 7) and body weight (*r*^*2*^_LHTV_ = 0.68; *r*^*2*^_TBST_ = 0.62; *r*^*2*^_DHBL_ = 0.72; *r*^*2*^_TTCM_ = 0.64; Fig. 8). This correlation was expressed through the regression equation LogF = a × LogW + b showing that the larger the fish, the more eggs were released.

## Discussion

The detection of stage IV and V oocytes in this fish during all months of the study showed that they were capable of spawning all year-round. In addition, the histological development in the adult stage of the gonads (stages IV and V) showed the appearance of various types of oocytes at the immature stage, such as O, PO, PVO, and SVO. Thereby it was seen that after the mature oocytes were released, the lower grade oocytes continued to develop and continue the cycle. It suggests that the reproductive form of this fish was spawning many times during the spawning season. This was considered a familiar property of the most economical fish species in the VMD. Some species belonging to this group include *Eleotris melanosoma* (Vo & Tran, 2014), *Oxyeleotris urophthalmus* (Vo, Tran & Mai, 2014), *Glossogobius giuris* (Hossain, 2014; Dinh et al., 2022b), *Butis butis* (Dinh & Le, 2017), *Stigmatogobius pleurostigma* (Dinh & Tran, 2018), *Periophthalmodon schlosseri* (Tran et al., 2019), *Glossogobius sparsipapillus* (Nguyen et al., 2019), *Periophthalmodon septemradiatus* (Dinh et al., 2020), and *Glossogobius aureus* (Dinh et al., 2021b).

Although *A. viridipunctatus* was capable of spawning year-round, the spawning season focuses on specific months. The primary spawning season of this fish showed different durations in the four study sites. The spawning season lasted for two months at TBST (June and July) and DHBL (August and September), whereas the spawning season at the other two sites was longer, with five months at LHTV (from June to October) and TTCM (from May to September). Thereby it was seen that in each different location, the fish had a spawning season adapted to the conditions of that area. In general, this fish species had a wet season spawning season in all study sites. Studies have found that some fish species exhibit a spawning season that starts early from the end of the dry season and lasts until the end of the wet season, such as *Glossogobius giuris* (Hossain, 2014; Dinh et al., 2022b), *Eleotris melanosoma* (Vo & Tran, 2014), and *Stigmatogobius pleurostigma* (Dinh & Tran, 2018). While in fish like *Pseudapocryptes elongatus* (Tran, 2008), *Oxyeleotris urophthalmus* (Vo, Tran & Mai, 2014), *Eleotris melanosoma* (Vo & Tran, 2014), *Butis koilomatodon* (Dinh et al., 2021a), and *Periophthalmodon schlosseri* (Tran et al., 2019) studies have shown a spawning season that lasts most months during the wet season (Table 3). *Glossogobius sparsipapillus* (Nguyen et al., 2021) and *Glossogobius aureus* (Dinh et al., 2021b) were two species found with a short spawning season of 2–3 months in the wet season like the results seen at TBST and DHBL. Not only do species in the VMD have a spawning season that occurs during the wet season, but some other fish species in the Gobiinae subfamily distributed worldwide also have a similar spawning season. *Gobius paganellus* distributed on the Isle of Man has a spawning season from mid-April to mid-June (Miller, 1961). *Glossogobius giuris* distributed in Payara River, Bangladesh has a spawning season from April to June (Qambrani et al., 2016). The same species, but distributed in Patuakhali, Bangladesh has a later spawning season in December (Roy et al., 2014). The spawning season of *Afurcagobius tamarensis* distributed in Murray Mouth and Coorong (Australia) takes place from October to December every year (Cheshire et al., 2013), whereas, *Padogobius martensi* in Italy has a spawning season believed to take place in May and June (Cinquetti & Rinaldi, 1987).

**Table 3:** Spawning season, the length at first maturity, and fecundity in some species.

Species	Spawning season	*L*_*m*_	Fecundity	References
*Gobius paganellus*	Mid-April to mid-June	–	–	Miller (1961)
*Padogobius martensi*	May and June	–	–	Cinquetti & Rinaldi (1987)
*Acentrogobius plaumi*	–	–	3,600–9,700	Baeck, Kim & Huh (2004)
*Afurcagobius tamarensis*	October to December	–	–	Cheshire et al. (2013)
*Acentrogobius* sp.	–	–	8,250	Soekiswo, Widyorini & Solichin (2014)
*Butis butis*	Year-round	6.82	46,017–78,500	Dinh & Le (2017)
*Stigmatogobius pleurostigma*	March to November	4.14	3,100–5,650	Dinh & Tran (2018)
*Periophthalmodon schlosseri*	Year-round	19.3–19.7	41,822–53,402	Tran et al. (2019)
*Periophthalmodon septemradiatus*	Year-round	6.05–7.23	5,916–11,451	Dinh et al. (2020)
*Glossogobius sparsipapillus*	July to September	6.50–6.78	17,918–28,700	Nguyen et al. (2021)
*Glossogobius aureus*	August to October	7.77–12.21	1,044–27,349	Dinh et al. (2021b)
*Glossogobius giuris*	April and in September	4.82–6.14	5,118–100,003	Dinh et al. (2022c)
*Acentrogobius viridipunctatus*	June to October in LHTV	6.6 ± 0.2 in LHTV	27,698 ± 7,983 in LHTV	This study
	June and July in TBST	7.5 ± 0.3 in TBST	46,592 ± 7,264 in TBST	
	August and September in DHBL	8.6 ± 0.2 in DHBL	23,271 ± 4,985 in DHBL	
	May to September in TTCM	9.4 ± 0.4 in TTCM	31,408 ± 2,515 in TTCM	

The length at first maturity in *A. viridipunctatus* varied by study area. This may be due to environmental effects and changes in each study site that have affected the *L*_*m*_ of fish (Dinh et al., 2021a). In this fish, *L*_*m*_ was found in areas of high salinity, such as DHBL. However, the temperatures at the study sites have similar values. That suggests that the temperature was not the factor that changed the *L*_*m*_ of fish. According to the salinity data collected at these four sites during the study months, the salinity changes were similar to the changes of *L*_*m*_. Specifically, in areas with high salinity, such as TTCM (28.7 ± 1.1‰) and DHBL (32.3 ± 1.0‰), *L*_*m*_ was significantly more prominent than in low salinity areas in TBST (19.8 ± 2.4‰) and LHTV (17.8 ± 2.3‰). This similarity showed that females in *A. viridipunctatus* had *L*_*m*_ that changes proportionally with salinity, revealing that this goby was observed to mature early at the site with higher salinity. Changes in *L*_*m*_ with salinity have been demonstrated in several other fish species. The *L*_*m*_ of *G. giuris* was adjusted to salinity variation as it matured earlier in saline areas (4.8 cm) than at the sampling sites with year-round freshwater conditions (6.1 cm) (Dinh et al., 2022c). In *Glossogobius aureus*, *L*_*m*_ tended to decrease from freshwater (12.5 ± 1.5 cm) to saltwater (10.5 ± 0.3 cm) (Dinh et al., 2021b). Similarly, in another species of mudskipper, *P. septemradiatus, L*_*m*_ increased from saltwater (8.2 cm) to freshwater (9.2 cm) (Dinh et al., 2020).

Fecundity is a characteristic factor for the development of each fish species. A high batch fecundity (*F*) was recorded in this fish with 5,481–130,683 eggs/female. This fish can easily repopulate with a high *F* with reduced numbers (McDowall, 1997). A species of the genus *Acentrogobius* was *Acentrogobius plaumi* in Korea, which showed relatively low fertility and ranged from 3,600 to 9,700 eggs (Baeck, Kim & Huh, 2004). Similarly, *Acentrogobius* sp. distribution in Indonesia also showed a low fecundity with 8,250 eggs (Soekiswo, Widyorini & Solichin, 2014) (Table 3). The results showed this fish species has a significantly higher reproductive capacity than other fish of the same genus. In addition, compared with some other fish species distributed in the VMD area, this fish has a significantly higher number of eggs. Some species with low fecundity can be mentioned as *Stigmatogobius pleurostigma* (3,100–5,650 egg/female) (Dinh & Tran, 2018), *Parapocryptes serperaster* (6,000–11,700 egg/female) (Dinh et al., 2016), *Trypauchen vagina* (4,000–12,750 egg/female) (Dinh, 2018), and *Periophthalmodon septemradiatus* (969–19,536 egg/female) (Dinh et al., 2020). Higher fecundity was observed in species such as *Pseudapocryptes elongatus* (2,100–29,400 egg/female) (Tran, 2008), *Boleophthalmus boddarti* (9,800–33,800 egg/female) (Dinh, Nguyen & Nguyen, 2015), and *Butis koilomatodon* 3,085–32,087 egg/female) (Dinh et al., 2021a). Moreover, *Glossogobius sparsipapillus* (8,568–95,191 egg/female) (Nguyen et al., 2021) and *Glossogobius giuris* (5,118–100,003 egg/female) (Dinh et al., 2022c) were two species of goby fish in the family Gobiidae shown to have a high fecundity and roughly equivalent to *A. viridipunctatus* (Table 3). In particular, *Glossogobius giuris* distributed in Kissorgonj, Bangladesh displayed fecundity up to 14,987–716,400 eggs/female (Hossain, 2014). These results showed that fish fecundity depends not only on species but also on their living environment.

## Conclusion

The results show the fish was a multi-spawner releasing eggs all year-round with a peak in the wet season. Fish size at first maturity varied by study site. It displayed a high fecundity that increased relatively with fish total length and body weight. In order to ensure sustainable exploitation, the fish should not be caught during the main spawning period, whilst the length at first capture should be >*L*_*m*_.

## Supplemental Information

10.7717/peerj.14077/supp-1 Supplemental Information 1 Raw data: Ovary *Acentrogobius viridipunctatus*.

## Additional Information and Declarations

### Field Study Permissions

The following information was supplied relating to field study approvals (*i.e*., approving body and any reference numbers): In terms of the field trip, our research has no field permit requirement as we collect fish from the public-owned where local fishers collect fish daily. Moreover, we collect fish just once a month at each site, and the number of fish collection is within allowable limits of the animal assessment.

### Data Availability

The following information was supplied regarding data availability: The raw measurements are available in the Supplemental File.
